# Prenatal Management of Congenital Human Cytomegalovirus Infection in Seropositive Pregnant Patients Treated with Azathioprine

**DOI:** 10.3390/diagnostics10080542

**Published:** 2020-07-30

**Authors:** Paolo Ivo Cavoretto, Chiara Fornara, Cristina Baldoli, Alessia Arossa, Milena Furione, Massimo Candiani, Patrizia Rovere Querini, Graziano Barera, Antonella Poloniato, Gerarda Gaeta, Arsenio Spinillo, Daniele Lilleri

**Affiliations:** 1 Obstestrics and Gynaecology Department, IRCCS San Raffaele Hospital and University, Via Olgettina, 60 20132 Milan, Italy; cavoretto.paolo@hsr.it (P.I.C.); candiani.massimo@hsr.it (M.C.); gaeta.gerarda@hsr.it (G.G.); 2 Laboratory of Genetics—Transplantation and Cardiovascular Diseases, Fondazione IRCCS Policlinico San Matteo, 27100 Pavia, Italy; c.fornara@smatteo.pv.it; 3 Neuroradiology Department, IRCCS San Raffaele Hospital, Via Olgettina, 60 20132 Milan, Italy; baldoli.cristina@hsr.it; 4 Obstestrics and Gynaecology, Fondazione IRCCS Policlinico San Matteo, 27100 Pavia, Italy; a.arossa@smatteo.pv.it (A.A.); a.spinillo@smatteo.pv.it (A.S.); 5 Microbiology and Virology, Molecular Virology, Fondazione IRCCS Policlinico San Matteo, 27100 Pavia, Italy; m.furione@smatteo.pv.it; 6 Autoimmunity and Gender Medicine Unit, Department of Internal Medicine, Division of Immunology, Transplantation and Infectious Diseases, IRCCS San Raffaele Hospital and University Via Olgettina, 60 20132 Milan, Italy; rovere.patrizia@hsr.it; 7 Neonatology and Pediatrics Departments; IRCCS San Raffaele Hospital, Via Olgettina, 60 20132 Milan, Italy; barera.graziano@hsr.it (G.B.); poloniato.antonella@hsr.it (A.P.)

**Keywords:** congenital cytomegalovirus infection, azathioprine, pregnancy, ultrasound, fetal MRI

## Abstract

Human cytomegalovirus (HCMV) is the leading infectious agent causing congenital disabilities. The risk of HCMV transmission to the fetus in pregnant women receiving immunosuppressive agents is unknown. We describe two cases of pregnant women with evidence of pre-conception HCMV protective immunity receiving azathioprine for ulcerative colitis or systemic lupus erythematosus. Both women reactivated the HCMV and transmitted the infection to the fetuses. One newborn showed unilateral hearing deficits and brain abnormalities while the other was asymptomatic. The mother of the symptomatic newborn had low levels of total and HCMV-specific blood CD4^+^ T cells. Women receiving immunosuppressive agents deserve information about the risk of HCMV congenital infection and should be monitored for HCMV infection during pregnancy. Their newborns should be screened for HCMV congenital infection.

## 1. Introduction

Human cytomegalovirus (HCMV) is the leading infectious agent causing congenital disabilities. About 0.6% of newborns are congenitally infected with HCMV, and about 20% of these are symptomatic at birth or develop long-term sequelae, in particular, hearing loss [1,2]. Maternal immunity has a role in preventing HCMV transmission to the fetus [3]; however, pre-conception immunity does not provide complete protection [4]. Pregnant women receiving immunosuppressive treatment may have an increased risk of HCMV reactivation and transmission to the fetus. However, the actual frequency of HCMV infection and its consequences in pregnant women treated with immunosuppressive agents is not known, and few reports exist on congenital HCMV infection in this setting [5,6,7,8]. Here, we describe two cases of congenital infection in pregnant women receiving azathioprine for ulcerative colitis (UC) or systemic lupus erythematosus (SLE). The study was approved by the Ethics Committee Pavia (proc. No. 20170011101, 10 April 2017).

## 2. Case Presentation

Case 1 was a 37-year old woman on her third pregnancy (one son) suffering from UC and receiving 100 mg/day of azathioprine. Due to a previous history of HCMV colitis, she was monitored for HCMV infection and immunity during pregnancy (Table 1). HCMV DNA became intermittently apparent in the blood from 17 weeks of gestation but was not detected in urine, saliva and vaginal secretions at the only time point examined (23 weeks). HCMV-specific IgG was present and IgM was absent throughout pregnancy. HCMV-specific CD4^+^ and CD8^+^ T cells were in the ranges of healthy controls [9]. Prenatal diagnosis was offered, but the patient refused invasive procedures. Detailed prenatal ultrasound examinations were all normal. A female child was delivered after 37 weeks (birthweight: 3050 g; Apgar scores: 9/9), and congenital HCMV infection was diagnosed by urine examination at birth. The child was asymptomatic at birth, with no sequelae at 4 years.

**Figure 1:**
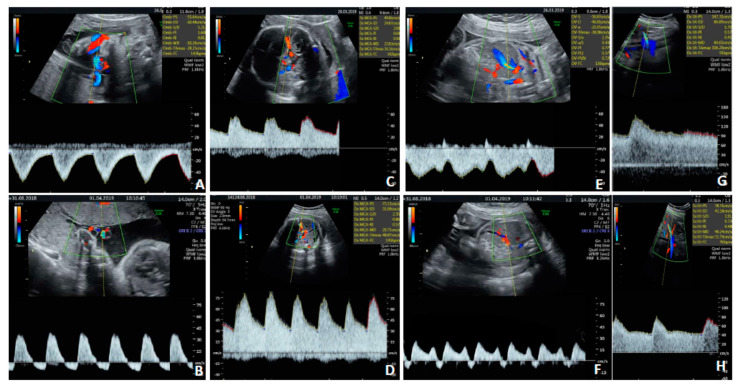
Prenatal Doppler findings for Case 2, showing the rapid progression of fetal Doppler alterations due to CMV infection. Umbilical artery Doppler at diagnosis of fetal growth restriction (**A**) and at delivery 4 days later (**B**). Middle cerebral artery Doppler at diagnosis (**C**) and at delivery (**D**). Ductus venosus Doppler at diagnosis (**E**) and at delivery (**F**). Uterine artery Dopplers were both normal (**G**,**H**).

**Figure 2:**
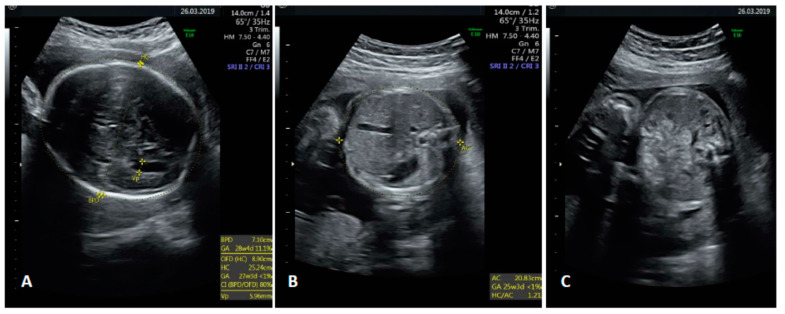
Fetal ultrasound findings for Case 2 at 30 + 3 weeks showing a normal brain in the transventricular plane (**A**), normal abdomen (**B**) and minor bowel echogenicity, consistent with the diagnosis of early onset fetal growth restriction (**C**). Neither hepatosplenomegaly nor other signs suggestive of fetal infection were detectable at that stage.

**Figure 3:**
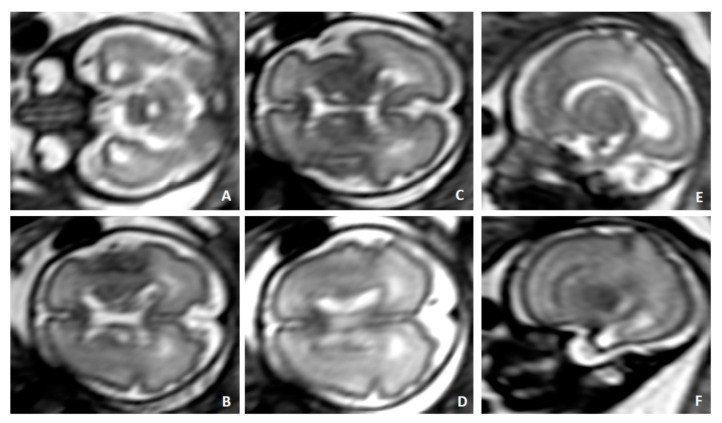
Fetal MRI, 30 GW of Case 2; SSHTSE (Single Shot Turbo Spin Echo) sequences and transverse (**A**–**D**) and sagittal images (**E**,**F**). Typical temporal pole abnormalities: temporal horn enlargement with cystic appearance (**A**–**F**), ventricular septation and white matter hyperintensity. Diffuse white matter hyperintensity. Multiple subependymal cysts.

Case 2 was a 38-year old woman on her second pregnancy (no offspring) suffering from SLE. She was receiving azathioprine at 50 mg/day, hydroxychloroquine at 200 mg every other day and acetylsalicylic acid at 100 mg per day. HCMV seropositivity was documented 1 year before pregnancy. Combined screening for major aneuploidies/preeclampsia and an anomaly scan were negative. An ultrasound (US) scan at 30 weeks showed fetal growth restriction with centralized circulation (Figure 1A,C,E,G) and isolated mild bowel echogenicity (Figure 2). Fetal brain magnetic resonance imaging (fbMRI) confirmed a pattern suggestive of fetal HCMV infection (Figure 3). After a standard course of steroids and magnesium sulphate, cesarean delivery was performed 5 days later due to the rapid deterioration of fetal wellbeing and Doppler studies (Figure 1B,D,F,H). The birthweight was 920 g; Apgar scores, 5/8; hemoglobin concentration, 14 g/dL; platelet count, 36,000/mL; and neutrophil count, 1000/mL. HCMV DNA was detected in maternal urine and vaginal secretions but not in the blood or saliva (Table 1). HCMV-specific IgM was absent at delivery and in a retrieved serum sample collected at 10 weeks, which showed undetectable HCMV DNA. At delivery, total CD4^+^ T-cell counts were low. More importantly, HCMV-specific CD4^+^ (but not CD8^+^) T cells were below the cutoff for healthy subjects (Table 1). HCMV DNA was detected in the blood, saliva and urine of the newborn along with HCMV-specific IgM.

**Figure 4:**
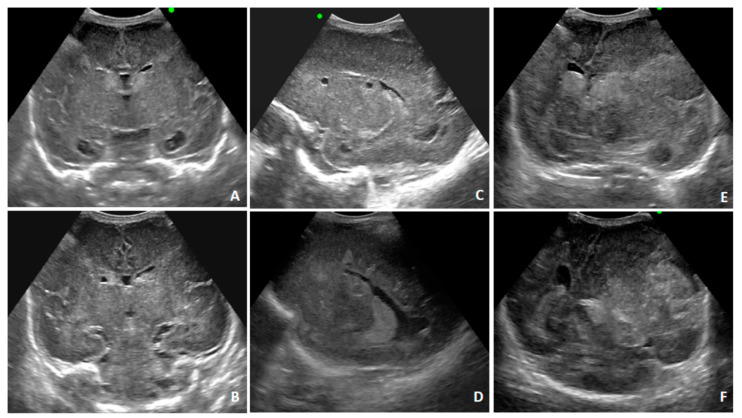
Transfontanellar ultrasound findings for Case 2 at birth. Second day after birth; (**A**–**D**): multiple subependymal cysts; temporal horn cysts. Bilateral microhemorrhagic alterations in the thalamic–caudate groove (germinal matrix). Diffuse white matter hyperechogenicity. Two days later; (**E**,**F**): bulky hemorrhagic lesions in left cerebral hemisphere with an extraparenchymal component appearing, causing an important mass effect and contralateral midline shift.

**Figure 5:**
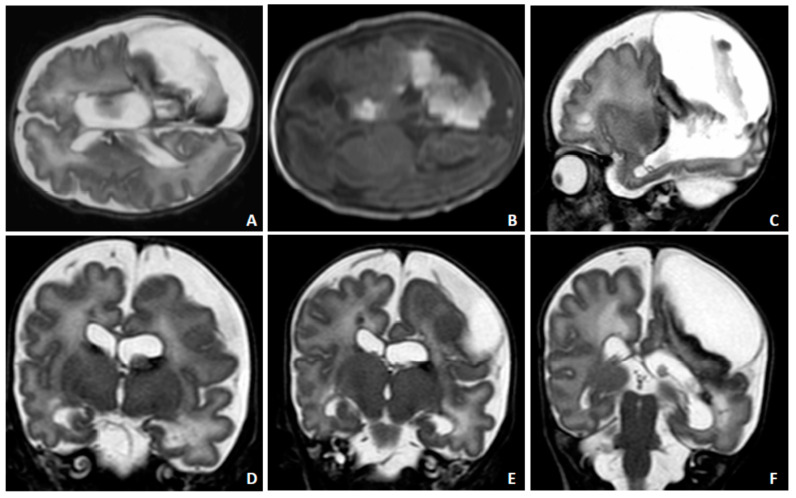
Neonatal MRI for Case 2 after 20 days of postnatal life. Chronic intraparenchymal and extraparenchymal hemorrhagic lesion with cystic appearance in the left cerebral hemisphere, ex vacuo ventricular enlargement (**A**–**F**) ((**A**,**B**): see paramagnetic component in T2 and T1 W images due to methemoglobin and hemosiderinic residual alterations). Persistent intraventricular septation with temporal horn cyst (**C**,**E**,**F**).

A postnatal transfontanellar US examination confirmed the alterations detected with fbMRI and diagnosed a microhemorrhage of the germinal matrix (Figure 4A–D). A control US examination three days later showed bulky hemorrhagic lesions (Figure 4E,F). A control fbMRI after 20 days highlighted the chronic evolution of the hemorrhagic alterations (Figure 5). The child was treated with ganciclovir followed by valganciclovir for five months. He is currently 14 months old and presents clinically relevant reduced somatic growth. A multidisciplinary follow-up was planned up to school age due to the high risk of future neurodevelopmental abnormalities.

## 3. Discussion

HCMV causes the most common congenital infections, and about 20% of infants infected in utero develop long-term sequelae such as hearing loss, mental retardation and psychomotor disabilities [1,2]. Prenatal screening and diagnosis are challenging [10,11], and prenatal treatment is difficult, despite recent evidence for the efficacy of biweekly hyperimmunoglobulin courses for primary infections [12].

Seronegative pregnant women caring for young children due to personal or occupational reasons are at higher risk of acquiring primary HCMV infection [13], which is transmitted to the fetus in 30–40% of cases. Congenital infection can also occur in seropositive women, although at a substantially lower rate [1,4,14]. Whether congenital infection in mothers with protective titers of HCMV-specific antibodies reflects a reactivation of the latent strain or a reinfection with a new strain in the mother remains undetermined, as does whether specific clinical or laboratory features might help in identifying patients at higher risk. A multicenter study (NCT03973359) is ongoing in Italy to define the actual frequency of HCMV congenital infections in pregnant women with pre-conception immunity and potential risk factors.

Here, we describe two prenatal cases of non-primary HCMV infection in HCMV-seropositive pregnant women receiving azathioprine for UC or SLE who delivered an HCMV-infected newborn. Case 1 had T-cell counts in the normal range [9] and delivered an asymptomatic newborn, whereas Case 2 showed a severe reduction in both total and HCMV-specific CD4^+^ T cells [9] and underwent iatrogenic preterm delivery. Her child was severely symptomatic due to growth restriction and brain abnormalities. The delayed development of the memory CD4^+^ T-cell response is associated with a higher risk for virus transmission to the fetus in pregnant women with primary HCMV infection [3]. In the two cases reported here, the more severe congenital infection occurred in the child of the woman with the documented impairment of the T-cell response.

In the literature, data on HCMV reactivation in pregnant women receiving immunosuppressive agents are scarce [5,6,7,8]. Awareness of the need for monitoring HCMV (and other opportunistic infections) during pregnancy in women receiving immunosuppression is greater among physicians attending to recipients of or candidates for organ or stem cell transplants [15]. An impairment of the T-cell response (especially CD4^+^) is associated with more severe HCMV infection in transplant recipients [16,17], and azathioprine-containing regimens are more likely to favor HCMV infection [18]. Immune-suppressive drugs may increase the risk of HCMV reactivation and transmission to the fetus during pregnancy and in other clinical conditions. We cannot exclude that, besides azathioprine treatment, other pre-conception potential risk factors could have contributed to HCMV transmission, such as the immune dysregulation induced by the underlying disease itself. However, all major additional causes of immune suppression were excluded in our patients (Table 2). The definition of the detailed pathogenesis of HCMV infection was outside of the scope of this report, but it opens the door to future studies on the clarification of the immune response to HCMV during pregnancy.

In Case 1, HCMV DNA was detected occasionally at different time-points. Case 2 was not monitored during pregnancy, but HCMV infection was suspected at 30 weeks due to US and MRI findings of progressive fetal growth restriction with brain abnormalities. Congenital HCMV infection was diagnosed at birth, and HCMV DNA was retrospectively tested for in maternal blood at delivery and in a stored serum sample collected at 10 weeks of gestation. HCMV DNA was not detected in the two samples tested (but serum is much less sensitive than whole blood for the detection of HCMV DNA). On the other hand, serologic determinations did not provide information about HCMV reactivation, since IgM was absent in both women, as was the detection of HCMV DNA in the blood. The simultaneous assessment of circulating HCMV-specific CD4^+^ T cells could have further added to the monitoring of the patient, possibly allowing the earlier identification of the event.

## 4. Conclusions

Our cases, along with a few previous reports, indicate that careful risk–benefit analysis should be performed for pregnant women receiving immunosuppression with agents such as azathioprine. If treatment cannot be discontinued, patients should receive multi-disciplinary counseling involving maternal–fetal medicine specialists, clinical immunologists and virologists about the potential risk for HCMV congenital infection. Additionally, the patient should be monitored for HCMV DNA in the blood (serology is not helpful for the diagnosis of reactivation) and, if possible, for an HCMV-specific T-cell response. Finally, the newborns of women receiving immunosuppression should be screened at birth for HCMV congenital infection, in order to allow clinical follow-up in the case of infection and the timely treatment of the potential sequelae. Multicenter prospective studies aimed at defining the risk of congenital infection in pregnant women treated with immunosuppressive agents are warranted.

## Figures and Tables

**Table 1:** Virological and immunological follow-up of human cytomegalovirus (HCMV) infection.

Case	Age	Parity	Weeks of Gestation	HCMV IgM	HCMV IgG	HCMV DNA Copies/mL				Total T Cells/µL of Blood			HCMV-Specific T Cells/µL of Blood *	
						Blood	Urine	Salivary Swab	Vaginal Swab	CD4	CD8	CD4/CD8 Ratio	CD4	CD8
#1	37	1	2	neg	pos									
			9			0								
			17			365								
			23							527	363	1.45	3.79	51.91
			27			0								
			31			0	0	0	0					
			33			30								
			36 (delivery)	neg	pos	60								
#2	38	0	10	neg	pos	0 ^§^								
			31 (delivery)	neg	pos	0	0	19	132	329	302	1.09	**0.26**	2.75

* positive response >0.4 cells/µL of blood; ^§^ tested on serum; bold response under cutoff.

**Table 2:** Risk factors for pre-conceptional immune suppression excluded for both patients of our study.

Use of steroids
HIV infection
Chemotherapy
Radiation therapy
Monoclonal antibodies
TNF-α inhibitors and cytokines
Splenectomy or other rare causes of asplenia
Sickle cell anemia
Bone marrow ablation
Organ transplant
Genetic diseases:
severe combined immunodeficiency chronic granulomatous disease common variable immunodeficiency immunoglobulin A deficiency
Diabetes mellitus
Renal failure
Malnutrition
Alcohol use
Drug use or abuse
Cigarette smoking
